# *Research:* The First Science Partner Journal

**DOI:** 10.1155/2018/1340806

**Published:** 2018-02-04

**Authors:** Tianhong Cui

**Affiliations:** Department of Mechanical Engineering, University of Minnesota, USA

The China Association for Science and Technology (CAST) and AAAS/Science are proud to be publishing the first batch of articles in Research, the inaugural Science Partner Journal. Research will publish fundamental research in the life and physical sciences as well as important findings or issues in engineering and applied science. The journal will initially publish original research, reviews, and editorials. Topics of particular interest include, but are not limited to, biology and life sciences, micro- and nano-science, artificial intelligence and information science, new energy studies, mechanical science and engineering, environmental science, emerging materials research, and robotics and advanced manufacturing.

The goal of Research is to publish high-quality papers on cutting-edge topics and to establish an international platform for academic exchange, collaboration, and innovation. We expect these published papers to make significant impact in the research community. Outside of the pages of this journal, CAST is also planning to organize an annual international conference on topics relevant to Research, first occurring in China and then moving to other countries in future years. With leading experts invited to be keynote speakers, scientists from all over the world will gather together at this conference to disseminate and exchange their new research achievements.

The editorial board of* Research*, led by Professor Tianhong Cui from the University of Minnesota as the international Editor in Chief, was established together with fifty-five experts from ten countries spread across the disciplines that the journal hopes to cover. The editorial board members are all leading experts in their research domain, including Charles Lieber from Harvard University, John A. Rogers and Yonggang Huang from Northwestern University. The Editor in Chief and the editorial board members will be responsible for a high-quality peer review of submitted articles. A group of four managing editors from CAST in Beijing along with individuals from the AAAS/Science Office of Publishing aid in the daily peer review process, production coordination, and marketing efforts for this new journal.

As the product of international collaboration itself, we know that the success of Research will be the result of the global community of scientists who read, cite, and work with this new journal as authors, editors, and reviewers. We hope that you enjoy reading these articles and future content, and consider submitting your paper to the journal. Please also reach out to the journal's Editorial Office at research@cast.org.cn if you would like to learn more about becoming a reviewer or being involved in the editorial board.

*Tianhong Cui*

